# Myeloid lineage skewing due to exacerbated NF-*κ*B signaling facilitates osteopenia in Scurfy mice

**DOI:** 10.1038/cddis.2015.87

**Published:** 2015-04-16

**Authors:** T H-P Chen, G Swarnkar, G Mbalaviele, Y Abu-Amer

**Affiliations:** 1 Department of Orthopaedic Surgery and Cell Biology & Physiology, Washington University School of Medicine, St. Louis, MO 63110, USA; 2 Department of Internal Medicine, Division of Bone and Mineral Diseases, Washington University School of Medicine, St. Louis, MO 63110, USA

## Abstract

Immune surveillance through Foxp3+ regulatory T cells plays a crucial role in bone homeostasis. Scurfy, the mouse model of autoimmune IPEX syndrome, bears a loss-of-function mutation in *Foxp3* that leads to multi-organ inflammation. Herein, we report that scurfy mice exhibit severe bone loss mediated by accelerated osteoclastogenesis. Mechanistically, Foxp3 deficiency results in the upregulation of NF-*κ*B in T helper cells through the loss of repressive Foxp3/NEMO interaction, thereby unleashing NF-*κ*B-mediated over-production of pro-osteoclastogenic cytokines. Flow cytometry analysis shows marked increase in lin^-^Sca-1^+^c-kit^+^ hematopoietic stem cells (LSK HSCs) and granulocyte/macrophage progenitors (GMPs) in bone marrow of scurfy mice with corresponding exacerbated osteoclastogenic potential, implying that osteoclast progenitors are affected at a very primitive stage in this disorder. Scurfy LSK HSCs exhibit greater sensitivity to M-CSF and contain abundant PU.1+ *Sf* LSK HSCs compared with WT. Accordingly, genetic or pharmacological inhibition of M-CSF or mTOR signaling, but not IL-17 signaling, attenuates osteoclastogenesis and osteopenia in scurfy. Thus, our study suggests that Foxp3 deficiency leads to osteopenia owing to dysregulated NF-*κ*B activity and subsequent cytokine-mediated hyper-proliferation of myeloid precursors, and positions the NF-*κ*B pathway as a potential target for therapeutic intervention for this disorder.

Skeletal homeostasis is primarily controlled by bone-resorbing osteoclasts and bone-forming osteoblasts.^1^ The former cells originate from hematopoietic lineage and require M-CSF and RANKL to differentiate into multi-nucleated bone-resorbing cells.^2, 3^ The differentiation factors are derived from several sources, chiefly mesenchymal cells and activated T cells, suggesting that bone homeostasis is regulated by various systems, including the immune network. Regulatory T cells (Tregs) are crucial for maintaining immune self-tolerance.^4^ The development and survival of these cells depend on the expression of the transcription factor Foxp3.^5^ The functional significance of this transcription factor in Tregs was recognized in humans with immune dysregulation polyendocrinopathy and enteropathy X-linked (IPEX) syndrome.^6^ These patients, similar to scurfy (*Sf*) mice, lack Tregs owing to mutations in the *Foxp3* gene, which leads to the development of fatal autoimmune hyper-proliferative disease.^7^ In contrast, forced expression of Foxp3 alleviates autoimmune responses, such as inflammatory bowel disease.^7, 8^

T-cell immune regulation of skeletal homeostasis has been widely studied owing to inflammatory bone disparities.^9, 10^ The initial direct evidence for such regulation was the identification of T-cell-derived RANKL, the primary osteoclast differentiation factor.^11, 12^ Subsequent studies identified IL-17, product of Th17 cells, as the principal potent cytokine that induces the osteoclastogenic factors RANKL, IL-1 and tumor necrosis factor *α*.^13^ On the other hand, strong evidence suggests that Th-cell-derived IFN*γ* and IL-4 are potent inhibitors of osteoclastogenesis.^14^ Likewise, potentiating Treg cells by forced expression of Foxp3 increased bone mass in mice by impairing osteoclast differentiation.^15^ However, additional cellular and molecular details have been offered to describe the mechanisms underlying T-cell regulation by Foxp3 and suppression of osteoclastogenesis. In this regard, it was reported that transcriptional (NFAT and NF-*κ*B),^16^ cell–cell interaction (via CTLA-4)^17^ and cytokine-dependent^18^ mechanisms contribute to this response.

To further elucidate the cellular and molecular mechanism underlying T-cell regulation of osteoclast differentiation, we examined the skeletal development of the *Sf* mouse in which Treg differentiation and function are dysregulated owing to *Foxp3* mutation. Our findings show that the skeleton of *Sf* mice deteriorates because of elevated osteoclastogenesis. This phenomenon appears to be the result of increased frequency of a myeloid sub-population, namely GMPs, that gives rise to osteoclasts. The high frequency of these progenitors was maintained by higher levels of M-CSF as depletion of this factor reversed the osteoporotic bone phenotype. We further provide evidence that these cellular changes are likely subsequent to hyper-activation of NF-*κ*B signaling in CD4^+^ T cells and GMPs, suggesting that Foxp3 is an endogenous regulator of NF-*κ*B-mediated inflammatory and osteoclastogenic factors.

## Results

### *Sf* mice exhibit bone loss

**Figure 1:**
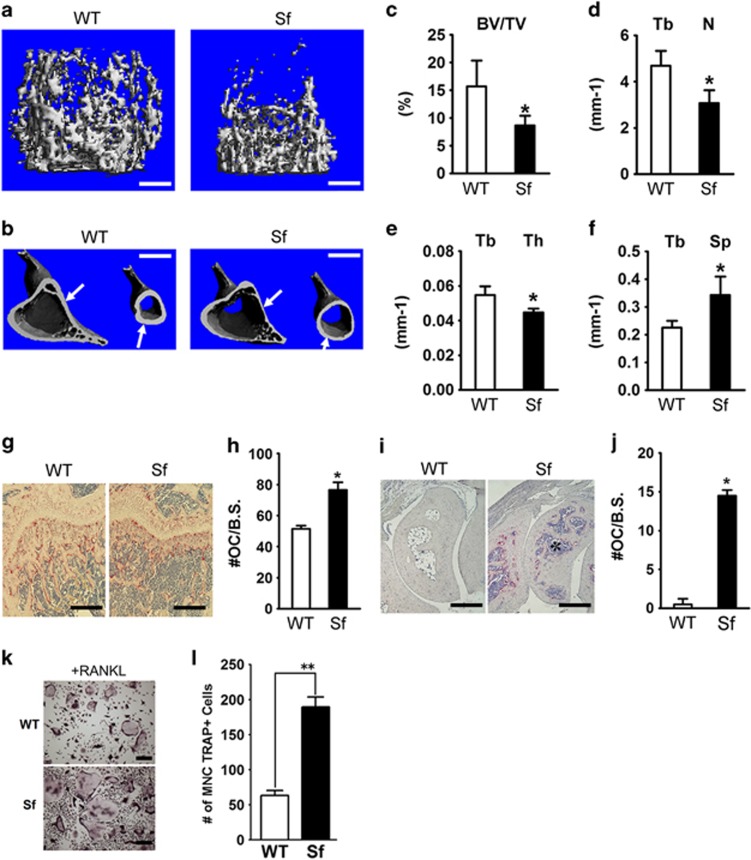
*Sf* mice exhibit severe bone loss and increased number of osteoclasts in bone. (**a**) Representative 3-D reconstructed images of the trabecular bone region in the proximity to the growth plate were captured by *μ*CT analysis. (**b**) Representative images of the proximal and midshaft cortex of the long bones are shown. (**c**–**f**) Quantification of bone parameters: (**c**) *μ*CT analysis depicts ratio of cancellous bone volume/tissue volume (BV/TV) in the same areas shown in panel **a**, (**d**) trabecular numbers (Tb N), (**e**) trabecular separation (Tb Sp), (**f**) and trabecular thickness (Tb Th). (**g**–**j**) Histological examination of osteopenia in *Sf* mice. (**g**) Representative H&E- and TRAP-stained histological section near the growth plate. (**h**) Quantifications of osteoclast number per surface area were plotted for the trabecular bone region. (**i**) Histological section of ankle bones. (**j**) Quantifications of osteoclast number per surface area were plotted for the sections near the ankle region. (**k**) WBM cultures from WT and *Sf* mice were subjected to vehicle or RANKL stimulation for 4 days before TRAP staining to visualize osteoclast formation. (**l**) Bar graphs were plotted by counting numbers of multi-nulceated (MNC) TRAP^+^ osteoclasts (OCs) from each of the 4 wells of each RANKL-stimulated groups. All bar graphs were depicted by the average of the numbers, ±S.D. Statistics: *n*=4 per group; *P<0.05 and **P<0.01 by Student *T*-test. Scale bars: 200 *μ*m in (**a**), (**i**) and (**k**); 1 mm in (**b**); 100 *μ*m in (**g**)

The effect of autoimmune response in many organs of *Sf* mice has been extensively studied.^19^ Given the pronounced inflammation-induced skeletal manifestations, we decided to investigate how lack of immune surveillance by Tregs in *Sf* mice affects bone. Bone morphometric and histological analyses on 4-week-old *Sf* as well as wild type (WT) littermates show significant reduction of bone volume in both trabecular (Figure 1a) and cortical (Figure 1b, arrows) bones of *Sf* mice. Quantitative analysis further indicated that all bone parameter changes (Figures 1c–f) denote bone loss in *Sf* mice. TRAP staining of histological sections of long (Figures 1g and h) and metatarsal bones (Figures 1i and j) revealed increased marrow cellularity (Figure 1i; asterisk) and elevated number of TRAP-positive cells per bone surface area in *Sf* mice (Figures 1h and j), suggesting that the bone loss phenotype of these mice is due to increased numbers of osteoclasts and their progenitors. Indeed, we established that *Sf*-derived whole bone marrow (WBM) cultures, plated at similar cell density to WT in the presence of RANKL and M-CSF, gave rise to increased number of osteoclasts compared with WT cultures (Figures 1k and l), which was also confirmed by semi-quantitative analysis of osteoclast marker expression (cathepsin K, MMP9 and *β*3 integrin) (Supplementary Figure S1A).

### NF-*κ*B activation and over-production of proinflammatory cytokines in *Sf*-derived T cells

**Figure 2:**
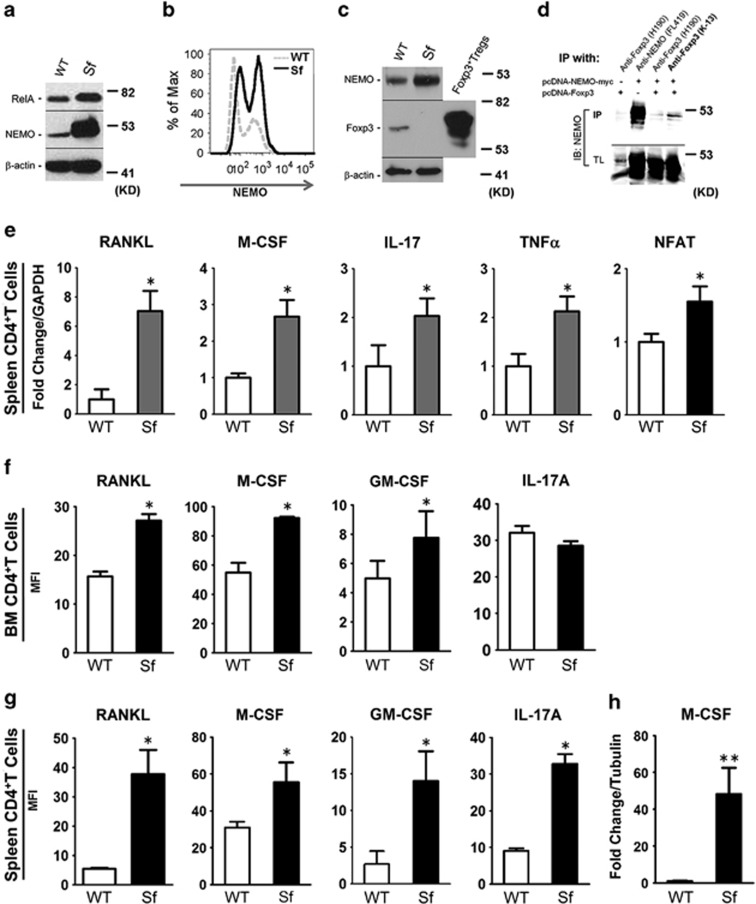
Foxp3 deficiency in *Sf* mice resulted in the activation of NF-*κ*B pathway and over-production of pro-osteoclast cytokines in CD4^+^ T lymphocytes. (**a**) Western blot of NEMO and Rel A protein expression in splenic CD4^+^ T cells using WT and *Sf*-derived CD4+ T cells. Images shown were blots probed with anti-Rel A and anti-NEMO Ab separately. *β*-actin was used as internal loading control. (**b**) Intracellular flow analysis was performed on red-cell-depleted WBMs. Cells were fixed, permeabilized and stained with anti-NEMO Ab followed by FITC-conjugated secondary Ab and analyzed on flow cytometer. Histogram of the middle fluorescence intensity (MFI) of FITC was shown. (**c**) Co-immunoprecipitation of Foxp3 and NEMO protein in primary splenic CD4^+^T cells. CD4^+^T-cell proteins derived from WT and *Sf* pulled down by anti-NEMO Ab were blotted with either anti-NEMO or anti-Foxp3 Ab. Total lysate from Foxp3^+^Tregs used as positive control. (**d**) Co-immunoprecipitation of Foxp3 and NEMO protein in transiently transfected 293 T cells. 293 T cells were transfected with both Foxp3 and NEMO (tagged with c-myc) plasmids. Cells transfected with either Foxp3 or NEMO plasmid alone were used as controls. Immunoprecipitation (IP) and immunoblots (IB) were conducted as indicated. In addition, two clones of anti-Foxp3 Ab were used for IP, by which K-13 generated stronger immunoprecipitation than H190. (**e**) qPCR analysis (fold change) of the indicated factors in MACS purified CD4^+^ T cells from WT and *Sf* mice. (**f**) Cytokine production following PMA stimulation was assessed by intracellular flow analysis on MACS-purified, BM-derived CD4^+^ T cells using appropriate antibodies. (**g**) Intracellular flow analysis on MACS-purified, PMA-stimulated spleen-derived CD4^+^ T cells. (**h**) qPCR analysis (fold change) of M-CSF expression in MACS-purified, anti-CD3/CD28 stimulated CD4^+^ T cells from WT and *Sf* mice. Bar graphs were depicted by the average of the numbers, ±S.D. Statistics for all bar graphs: *n*=3 per group; *P<0.05 and **P<0.01 by Student *T*-test

The transcription factor NF-*κ*B is considered the master regulator of inflammatory responses as it modulates the expression of a wide range of inflammatory cytokines, besides being also essential for osteoclastogenesis.^20^ We tested protein and mRNA expression of NF-*κ*B genes, and found that the expression of RelA/p65 and IKK*γ*/NEMO, the central modulators of NF-*κ*B signal transduction pathway, was predominately upregulated 2.1- and 5.6-fold for protein and 1.8- and 1.6-fold for mRNA, respectively (Figure 2a and Supplementary Figures S1B and S1C). Using intracellular flow assay, we found that the proportion of NEMO-expressing cells was distinctively increased in *Sf* bone marrow (Figure 2b, solid line). Further examination of NEMO-expressing cells by multicolor flow cytometry revealed that a subset of myeloid cells (CD11b^+^c-kit^+^) bore high NEMO expression (Supplementary Figure S2A). In addition, analysis of NEMO-expressing cells by CD11b and Gr1 resulted in the enrichment of CD11b^+^Gr1^-^ population by more than 2.5-fold (Supplementary Figure S2B). These findings implied the importance of NEMO upregulation in the myeloid proliferative disease in *Sf* mice. Using co-immunoprecipitations from primary CD4^+^ cells (WT and *Sf*) and from transiently transfected 293T cells, we show that NEMO and Foxp3 physically interact (Figures 2c and d). Further, using NF-*κ*B reporter assay, Foxp3 protein was able to suppress the reporter gene luciferase by 30% (Supplementary Figure S3A). In addition, increased NEMO expression was also found in the lymphoid tissues, including lymph nodes and thymus, but not in the peritoneal cavity, which was mostly comprised of macrophages (Supplementary Figures S3B–S3F). We next tested mRNA expression of pro-inflammatory/osteoclast cytokines in magnetically isolated T helper (T_H_) cells. As shown in Figure 2e, RANKL, M-CSF, IL-17 and tumor necrosis factor *α* were all found to be upregulated in *Sf*-derived CD4^+^ T_H_ cells, which were also more activated than the WT cells as evidenced by increased expression of the transcription factor NFAT. Intracellular flow assay further confirmed that both bone marrow (Figure 2f) and spleen-derived (Figure 2g), PMA/ionomycin-stimulated CD4^+^ T_H_ cells produced significantly higher levels of RANKL, M-CSF and GM-CSF, with spleen (but not bone marrow)-derived CD4^+^ T_H_ cells also producing higher IL-17. Similarly, when spleen-derived CD4^+^ T_H_ cells were stimulated with anti-CD3/CD28 antibody (Ab) beads, which is more physiologically relevant, we observed 48 × more expression of M-CSF in *Sf*-derived cells compared with WT controls (Figure 2h). Accordingly, multiplex ELISA showed elevated serum levels of a wide range of chemokines, inflammatory and growth factors in *Sf* mice compared with WT controls (Supplementary Figure S4). Taken together, Foxp3 deficiency resulted in the loss of Foxp3–NEMO interaction and led to the overexpression of NEMO and subsequent activation of NF-*κ*B in T_H_ cells. These pro-inflammatory cytokine-secreting T cells were hyperactivated, thereby were suspected as culprit inducers of higher osteoclastogenesis in *Sf* mice.

### Enhanced myelopoiesis/myeloproliferative disease in the *Sf* bone marrow leads to increased osteoclastogenic potential

**Figure 3:**
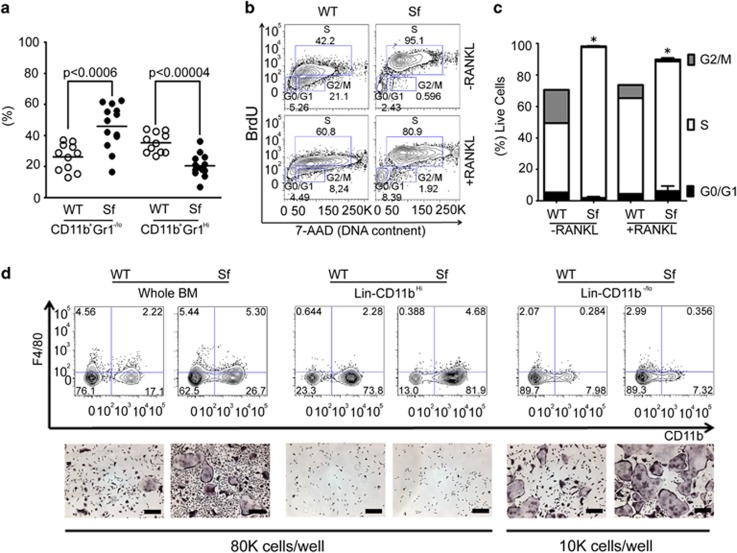
Bone marrow CD11b^-^, not CD11b^+^, cells from *Sf* are highly proliferative and osteoclastogenic. (**a**) Flow analysis was performed on bone marrow myeloid populations from 12 *Sf* and 11 WT controls stained with anti-CD11b and anti-Gr1 antibodies. Statistics was generated by Student *T*-test. (**b**) BrdU incorporation in *Sf* WBM cultures was carried out as described under methods. (**c**) Bar graph derived from BrdU flow assays shown in panel **b**. *n*=4 per group; *P<0.05 by Student *T*-test. (**d**) MACS was performed first with biotin-conjugated lineage antibodies and anti-biotin microbeads followed by anti-CD11b microbeads to obtain lin^-^CD11b^-/lo^ and lin^-^CD11b^Hi^ fractions. Osteoclatogenic potential of each MACS fraction was subsequently tested by *ex vivo* osteoclastogenesis assay. WBM cultures were used as control. Scale bars: 200 *μ*m in (**d**)

Next, we interrogated whether the increased osteoclast burden and osteopenia in *Sf* mice is due to skewed myelopoeisis secondary to hypercytokinemia. Because osteoclasts differentiate from the myeloid lineage, we hypothesized that dysregulation of lineage commitment or differentiation of myeloid progenitor cells significantly contribute to increased osteoclastogenic potential of *Sf* bone marrow. To test this, we performed flow cytometric analysis of the myeloid compartment in the bone marrow, the spleen and blood using monocytic marker CD11b and granulocytic marker gr1. As shown in Figure 3a, the percentage of monocytic CD11b^+^gr1^-/lo^ cells was significantly increased in bone marrow, whereas percentage of granulocytic CD11b^+^gr1^Hi^ cells was decreased. We further examined the proliferative activity of *Sf*-derived cultures using BrdU flow assay following 3 days of culture. *Sf* cultures exhibited nearly twice of BrdU incorporation (Figures 3b and c) and proliferation of *Sf* cells was much faster than WT cells in the absence (Supplementary Figure S5A) or presence of RANK ligand (Supplementary Figure S5B). Taken together, increased osteoclast formation in *Sf* mice is likely because of hyperproliferative osteoclast precursor cells in *Sf* mice.

Through separating CD11b^hi^ population from CD11b^lo^ after lineage depletion (i.e., T and B cells, erythroid), we found that CD11b^lo^ population exhibited far greater osteoclastogenesis potential than CD11b^hi^ population (Figure 3d). In addition, CD11b^lo^ population isolated from *Sf* bone marrow possessed hyperactive potency for osteoclastogenesis. In fact, almost an equal level of osteoclast formation could be achieved by plating 10 000 cells of CD11b^lo^ cells to 80 000 WBM cells (Figure 3d; compare right with left panels, respectively). We have also tested potential contribution of myeloid-derived suppressor cells (MDSCs) to the *Sf* osteopenic bone phenotype, as reported in murine breast cancer model, wherein tumor-associated MDSCs, marked as CD11b^+^Gr-1^+^ cells,^21^ and MDSCs derived from murine inflammatory arthritis model^22^ can be induced into osteoclasts. We found that *Sf* bone marrow contained slightly less frequency of Gr-1^hi^Ly6G^+^ MDSCs and much higher frequency of Gr-1^dim^Ly6G^-^ MDSCs (Supplementary Figure S6A). However, both cell populations exhibited poor osteoclastogenic potential (Supplementary Figures S6B and C) as compared with WBM cultures (Supplementary Figure S6D) and cell fractions devoid of the two MDSC populations (Supplementary Figure S6E). Taken together, although all immature myeloid cells were hyperproliferative in *Sf* compared with WT mice, only CD11b^lo/-^ cells exhibited high osteoclastogenic potential.

### Alteration of hematopoietic progenitor populations in *Sf* mice

**Figure 4:**
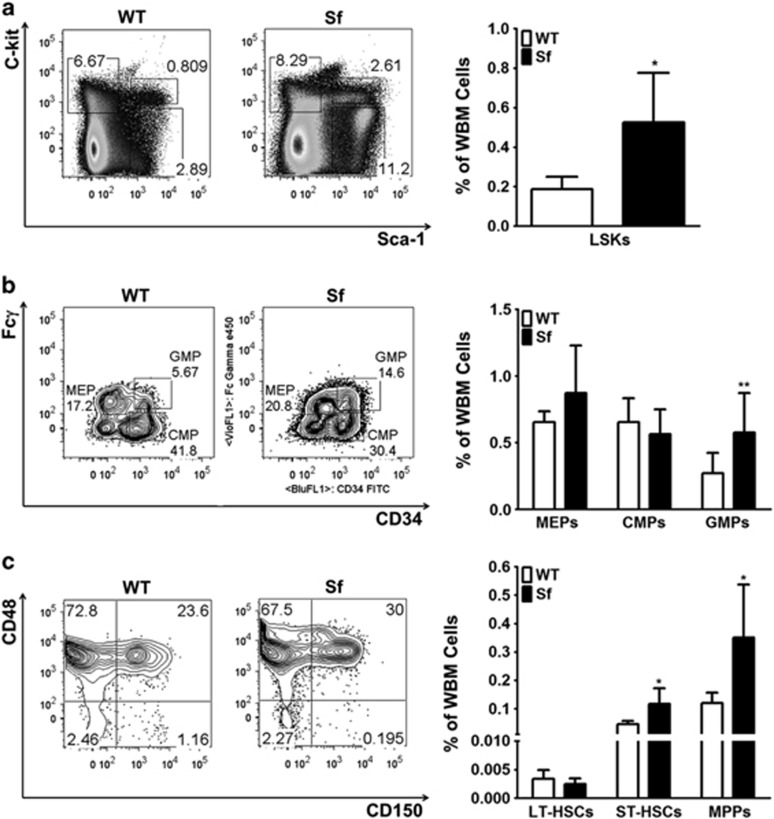
Alteration of hematopoietic progenitor pool in *Sf* bone marrow. (**a, b**) Flow cytometry of HSC progenitors was performed as described in Material and Methods. Percentage of LSK hematopoietic progenitor cells were first gated and analyzed, followed by further sub-gating of the lin^-^c-kit^+^ cell population (LK) into megakaryocyte/erythroid progenitors/CMP/GMPs. (**c**) LT-HSC, ST-HSCs and mutipotent progenitors were sub-gated from LSKs for analysis. Bar graphs were depicted by the average of the numbers, ±S.D. Statistics for all bar graphs: *n*=6 for WT group and *n*=7 for *Sf* group; *P<0.05 and **P<0.01 by Student *T*-test

To test how the number of myeloid progenitor cells is altered in *Sf* mice, we generated a comprehensive profile of myeloid/osteoclast progenitors from the most primitive (i.e., long-term or LT-HSCs) to the more committed (i.e., GMPs) cells.^23^ Frequency of the early lineage precursors lineage-negative, Sca-1-postive and c-kit-positive (LSK-HSCs) cells was significantly elevated in *Sf* compared with WT mice (Figure 4a). Among the more committed myeloid precursors, frequency of GMPs derived from *Sf* bone marrow was twice as much as from WT counterparts, whereas the frequency of other progenitors was not significantly changed (Figure 4b). Further characterization of LSKs by the SLAM marker (CD150) revealed a significant increase for both short-term HSC (ST-HSC) and mutipotent progenitor frequency, with greater change for the latter (Figure 4c). Taken together, these results suggested that under the inflammatory condition of *Sf* bone marrow, myelopoiesis was skewed toward granulocyte/macrophage lineage. The primitive hematopoietic progenitor cells at the stage as early as the ST-HSCs respond to the inflammatory cues by moving from quiescence to active cell division, resulting in increasing numbers of myeloid/osteoclast precursors. This phenomenon appeared to be lineage-specific, as GMP frequency observed in *Sf* bone marrow did not seem to be at the expense of megakaryocyte/erythroid progenitors.

### *Sf* LSK HSCs are potent osteoclast founder cells

**Figure 5:**
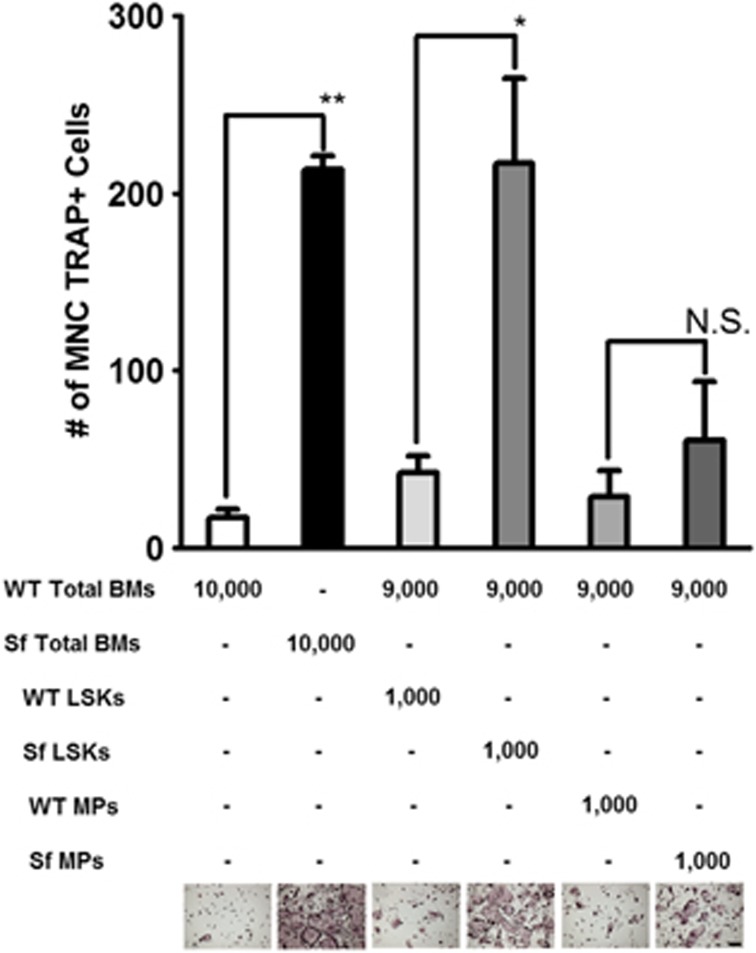
*Sf* LSK HSCs are potent osteoclast founders. One thousand FACS-sorted LSK and MPs (lin^-^c-kit^+^CD11b^lo^ described in Supplementary Figure S4) from WT and *Sf* bone marrows and mixed with 9000 total bone marrow cells from WT and treated with RANKL. WBM cultures were used as control. Bar graph was depicted by the average of OC numbers, ±S.D. Statistics; *n*=3 per group; *P<0.05 and **P<0.01 by Student *T*-test. Scale bar: 200 *μ*m

Although the number of osteoclast progenitors is significantly higher in *Sf*, the osteoclastogenic potential of each progenitor cell may be also exacerbated. Hence, we examined whether myeloid progenitors derived from *Sf* mice possess higher 'osteoclast founder' potential. Therefore, we FACS-sorted lin^-^c-kit^+^CD11b^lo^gr-1^lo^ cells (MPs) and LSK cells from WT and *Sf* mice (Supplementary Figure S7) and combined equal number of each cell population them with total bone marrow cells from WT at the ratio of 1 : 10 (Figure 5) or 1 : 5 (not shown) to make the culture with 10 000 and 5000 cells per well density, without or with RANKL. Total bone marrow cultures served as controls. As shown in Figure 5, WT-derived LSKs supplemented with WT WBMs resulted in a mild increase of osteoclastogenic potential, whereas *Sf*-derived LSKs supplemented with WT WBMs exhibited higher osteoclastogenic potential comparable with *Sf* WBMs. These data indicate that *Sf* LSK HSCs were indeed highly potent 'founders' for osteoclastogenesis. Together, these findings indicate that the ontology of increased osteoclastogenic potential came at an early and primitive stage of differentiation and commitment of *Sf* BM HSCs.

### Inhibition of M-CSF significantly rescued bone erosion in *Sf* mice

At the mechanistic level, this observation correlates with a recent report suggesting that HSCs can be a direct target for cytokine signaling.^24^ We reasoned that *Sf*-derived HSCs might be hypersensitive to M-CSF, one of the essential cytokines for osteoclast development. LSK HSCs were FACS-sorted, plated for osteoclastogenesis assay at 2000 cells per well and stimulated with serial concentrations of M-CSF. As shown in Supplementary Figure S8A, *Sf* -derived LSKs responded to M-CSF in a much greater manner than the WT controls. Furthermore, detection of PU.1 (which is the target of M-CSF signaling and crucial for osteoclastogenesis) expression in LSKs via intracellular flow revealed that its protein level is significantly increased in *Sf* LSKs (Supplementary Figure S8B), confirming the activation of M-CSF signaling cascade in these progenitor cells.

**Figure 6:**
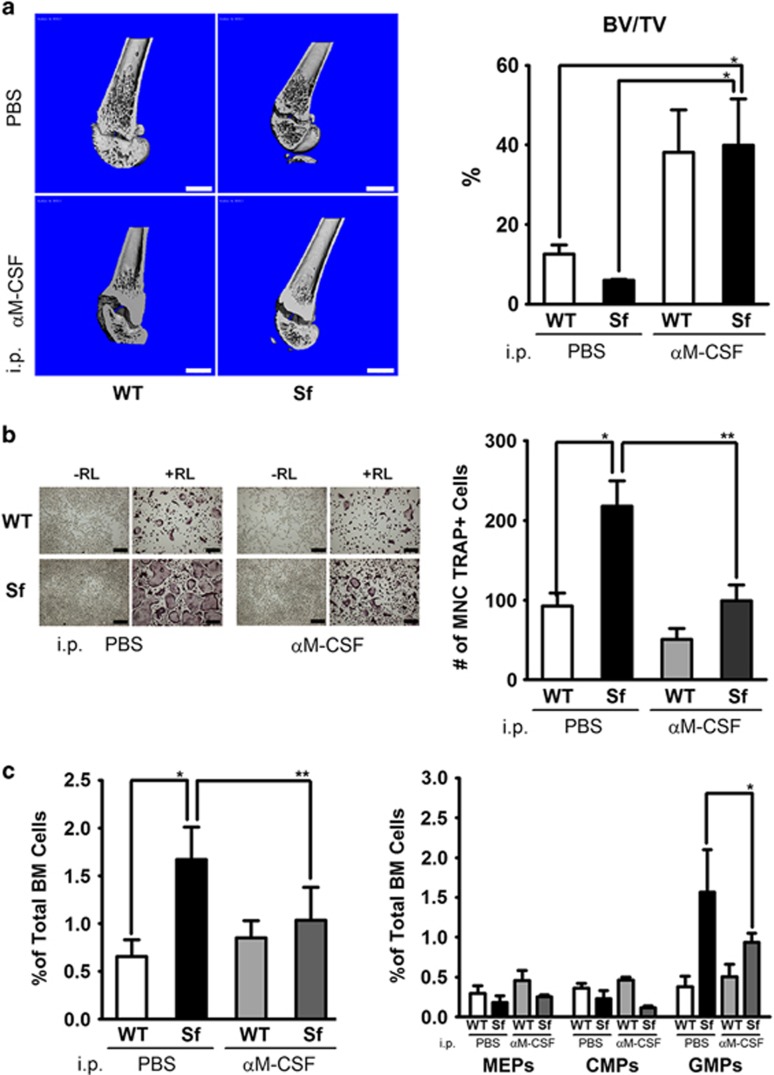
Neutralization of M-CSF significantly rescued osteopenia in *Sf* mice. (**a**) *Sf* and WT mice were injected with M-CSF-neutralizing Ab or PBS as described in Material and Methods. Cross-sections of femur bone extending from the growth plate were generated by 3-D reconstructed images through *μ*CT analysis. BV/TV ratios were depicted as bar graph. (**b**) *Ex vivo* osteoclastogenesis assay post *in vivo* administration of M-CSF-neutralizing Ab. (**c**) Flow cytometric analysis of WBM hematopoietic progenitors were performed on WBMs derived from M-CSF Ab-administrated *Sf* mice with the rest of experimental control mice. Statistics for all bar graphs: *n*=3 for WT group and *n*=4 for *Sf* group; *P<0.05 and **P<0.01 by Student *T*-test. Scale bars: 1 mm in (**a**); 200 *μ*m in (**b**)

To investigate the contribution of M-CSF hyper-production in osteo-pathogenesis of *Sf* mice, we administrated M-CSF-neutralizing Ab or carrier solution into *Sf* mice and WT male siblings for 2 weeks. As expected, WT mice exhibited osteopetrosis upon anti-MCSF Ab treatment assessed by *μ*CT analysis (Figure 6a). Interestingly, anti-M-CSF Ab-treated *Sf* mice also developed increased BV/TV value comparable with WT mice. *Ex vivo* osteoclastogenesis assay further demonstrated the rescue of osteopenia/hyper-osteoclastogenesis in *Sf* via M-CSF neutralization (Figure 6b). In fact, osteoclastogenesis derived from anti-MCSF-treated *Sf* mice was reduced to levels comparable with that derived from WT mice (Figure 6b; right panel). Interestingly, as anti-M-CSF Ab treatment significantly reduced the frequency of LSKs in *Sf* bone marrow, the frequencies of GMPs and mutipotent progenitors were moderately decreased (Figure 6c and Supplementary Figure S9). In addition, *ex vivo* osteoclastogenesis of *Sf* WBMs post M-CSF Ab treatment was significantly blocked.

### M-CSF haploinsufficiency significantly rescued the osteopenic phenotype of *Sf* mice

**Figure 7:**
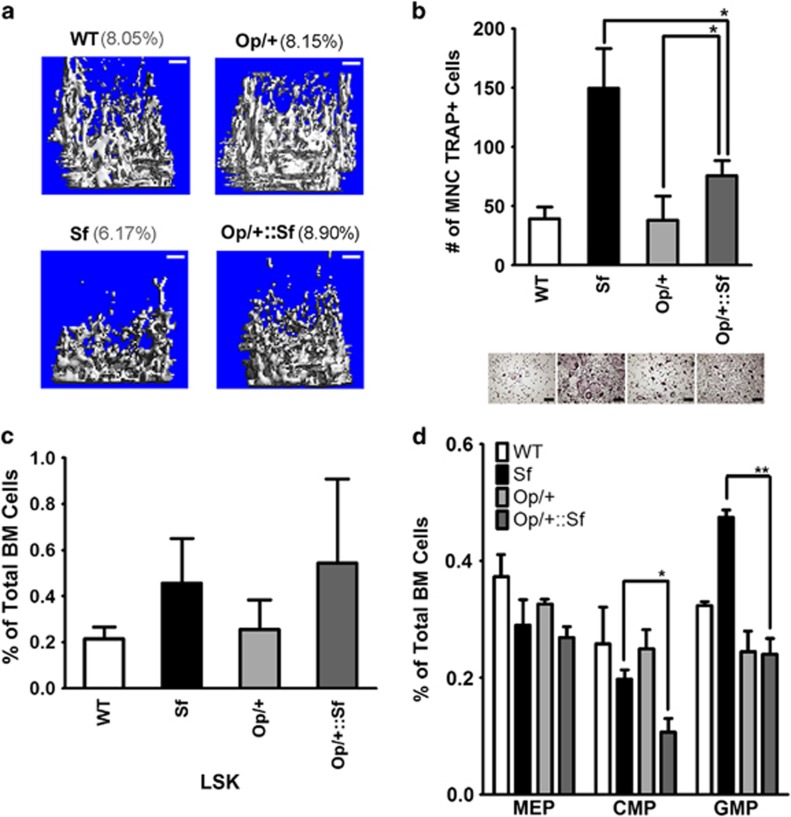
Rescue of the osteopenic phenotype in *Sf* via genetic ablation of M-CSF. (**a**) Representative *μ*CT scanned 3-D images of the femur trabecular area of WT, *Sf* and *Op/+:: Sf* mice. (**b**) *Ex vivo* osteoclastogenesis assay were performed using WBMs from WT, *Sf*, *Op*/+ and *Op*/+::*Sf* mice. (**c**) Frequency of LSK HSCs in Op/+ *Sf* mice using flow cytometry. (**d**) Profile of megakaryocyte/erythroid progenitors/CMP/GMPs of WBMs derived from WT, *Sf*, *Op*/+ and *Op*/+ *Sf* mice, *P<0.05 and **P<0.01 by Student *T*-test. Scale bars: 200 *μ*m in (**a** and **b**)

To further address the contribution of M-CSF signaling in the osteopenic phenotype of *Sf*, we crossed *Sf* hemizygous female (*Sf*/x) with Op heterozygous (*Op*/+) male to generate *Op*/+::*Sf* mice and asked how osteoclast progenitors derived from *Sf* would respond to M-CSF happloinsufficiency. As shown in Figure 7a, *μ*CT analysis revealed that introduction of one *op* allele in *Sf* mice (i.e., *Op*/+, *Sf*/y) was able to bring the trabecular BV/TV value equivalent to the WT control. In addition, WBMs derived from *Op*/+::*Sf* mice only gave rise to half osteoclast formation in number as compared with cells derived from *Sf* alone (Figure 7b). Interestingly, although the frequency of LSK HSCs did not change significantly (Figure 7c), frequency of CMPs and GMPs were reduced in *Op*/+::*Sf* (Figure 7d), suggesting that commitment or fate determination of HSCs in *Sf* bone marrow was altered by haploinsufficiency of M-CSF, resulting in attenuation of hyper-osteoclastogenic activity.

**Figure 8:**
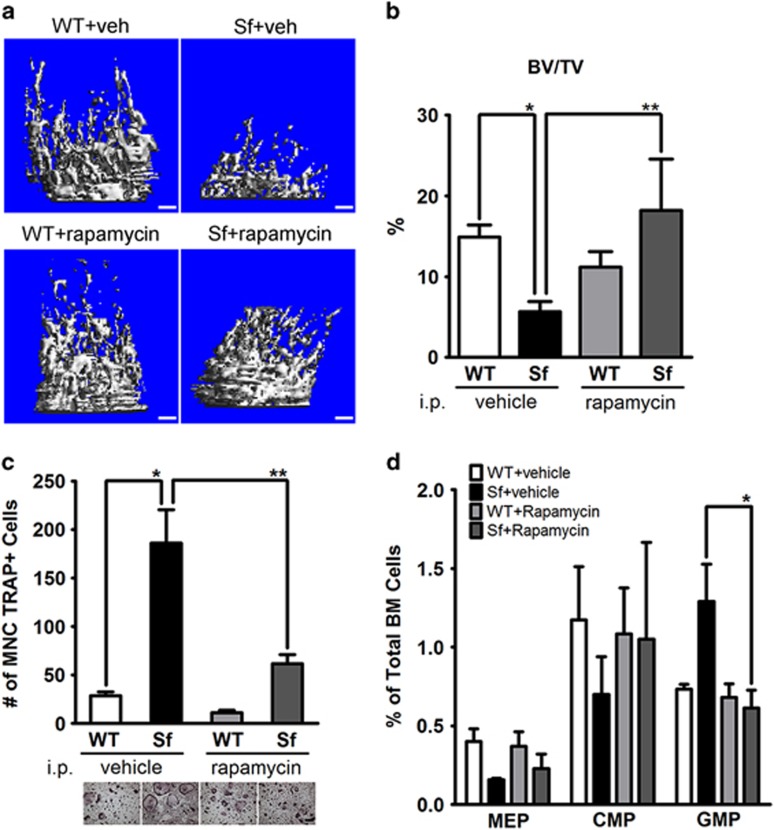
*In vivo* administration of rapamycin ameliorated osteopenia in *Sf* mice through reduction of osteoclastogenic activity in the bone marrow. (**a**) Representative images from *μ*CT analysis of rapamycin *versus* vehicle-treated *Sf* mice. (**b**) Trabecular BV/TV values of vehicle- and rapamycin-treated mice. (**c**) *Ex vivo* osteoclastogenesis assay. (**d**) Hematopoietic progenitor cell profiling by flow cytometry depicting megakaryocyte/erythroid progenitors, CMP and GMP cells from the various experimental groups described in panel **a**. Statistics for all bar graphs: *n*=4 for per group; *P<0.05 and **P<0.01 by Student *T*-test. Scale bars: Scale bars: 200 *μ*m in (**a** and **c**)

To probe the therapeutic value of targeting M-CSF, we tested whether blocking mTOR signaling (which is downstream M-CSF) would counteract the osteopenic disease in *Sf* mice. Indeed, following 2 weeks of intraperitoneal rapamycin injections, bone parameter of rapamycin-treated *Sf* mice was comparable with that of vehicle-treated WT controls (Figures 8a and b). Consistently, *in vivo* rapamycin treatment reduced osteoclastogenesis activity of *Sf*-derived bone marrow cells by almost twofold (Figure 8c). Interestingly, the frequency of GMP in *Sf* BM was significantly reduced to the level as in vehicle-treated WT controls (Figure 8d). Taken together, inhibition of mTOR pathway appeared to an effective approach on ameliorating *Sf*'s osteopenic phenotype.

## Discussion

In this study, we report severe osteopenia in *Sf* mice subsequent to the action of Foxp3-deficient lymphocytes. Mechanistically, we provide evidence that Foxp3 deficiency leads to enhanced activity of NF-*κ*B, exemplified by elevated expression of RelA/p65 and NEMO, the scaffold and regulator of NF-*κ*B signaling. As a result, numerous osteotropic and osteoclastogenic factors are produced, which mobilize hematopoietic progenitors pools, culminating with hyper-myeloproliferation of osteoclast progenitors, exacerbated osteoclastogenesis and osteopenia.

In recent years, several reports have suggested that Treg cells play an inhibitory role in osteoclast formation.^25, 26^ These studies focused on the interaction between Treg cells and osteoclast precursors, either through direct cell–cell contact or paracrine mechanisms. In this report, we provide evidence that lack of immune surveillance, due to Treg cell defeciency, leads to hyperactivation of effector immune cells. Specifically, we identified exuberant expression of RelA/p65 and NEMO as markers of hyperactivated NF-κB in pro-inflammatory cytokine-producing T effector (Foxp3-deficient) cells. Subsequently, production of pro-inflammatory cytokines is enhanced and in turn stimulates repopulation and/or renewal of hematopoietic progenitors. The cytokines produced by the T effector cells directly target hematopoietic stem cell pools and accelerate proliferation, mobilization and differentiation of myeloid progenitors into osteoclasts. This is further evident by the activation of NF-*κ*B in OC progenitors, which is a crucial osteoclastogenic factor. Indeed, inhibition of NF-*κ*B signaling by administration of NBD peptide halted osteoclastogenesis by *Sf*-derived progenitors (data not shown).

More importantly, through Ab neutralization and genetic ablation, we identified the critical role of M-CSF signaling rather than IL-17 in this process. We demonstrate that the commitment of HSCs is driven toward the macrophage/preosteoclast lineage and ultimately results in increased osteoclast formation and osteopenia. Our findings depict important changes to characteristics of osteoclast progenitors in the absence of immune surveillance. Specifically, LSK HSCs derived from *Sf* bone marrow were highly potent precursors and founders of osteoclasts. This finding can be illustrated as more osteoclast precursors are present in *Sf* LSKs and/or osteoclast precursors in the LSK pool are more sensitive to osteoclastogenic stimuli. This is further supported by our observations that more PU.1 LSKs and more GMPs were present in *Sf* bone marrow. Interestingly, the pharmacologic and genetic approaches we conducted here to inhibit the M-CSF/PI3K/mTOR axis markedly reduced the frequency of GMPs in *Sf* bone marrow. These signals, as our findings suggest, target the very primitive stage of hematopietic progenitor cells.

The apparent myeloproliferation and abundance of osteoclast progenitors is consistent with higher expression of M-CSF in *Sf* *mice*. This factor is obligatory for proliferation and survival of osteoclast precursors, and genetic ablation of the corresponding csf gene or its receptor c-fms obliterates osteoclastogenesis and leads to osteopetrosis.^27^ Our findings are supported by a recent report that demonstrated that under inflammatory conditions, high expression of M-CSF induces the myeloid master regulator PU.1 leading to changes in HSCs cell fate and skewing myeloid differentiation.^24^ Hence, modulating this pathway in *Sf* mice using pharmacologic and genetic approaches relieved the osteoclastogenic burden and significantly attenuated osteopenia in *Sf* mice. At the cellular level, these M-CSF inhibitory approaches clearly attenuated the increased frequency of the LSK and GMP cells in *Sf* mice leading to decreased osteoclastogenesis.

M-CSF activates mTOR/PI3K/Akt pathway, and it has been shown that rapamycin blocks M-CSF and RANKL-induced osteoclastogenesis.^28^ Our study offers novel insights pointing to inhibition of frequency of osteoclast progenitor pool by rapamycin in *Sf* mice leading to amelioration of osteopenia. Our findings suggest that mTOR signaling plays a key role in mediating bone loss in *Sf* mice and highlight the significance of M-CSF/mTOR/PI3K axis in autoimmune-mediated osteolytic diseases. This concept is further supported by recent evidence demonstrating significance of mTOR signaling in joint destruction via robust osteoclast activity,^29^ thereby positioning M-CSF signaling through mTOR as a significant target to combat immune-mediated bone destruction.

## Materials and Methods

### Mice

C57/B6 WT, *Sf* and Op mice were obtained from Jackson Laboratories (Bar Harbor, ME, USA). All mice were housed in CSRB NT or BJCIH animal facilities under the guidelines of the Animal Studies Committee at Washington University School of Medicine.

### Structural and quantitative analysis of bone deformities

Long bones were dissected from animals and fixed in 10% neutral buffered formalin for 2 days followed by three washes in PBS. Then muscle and connective tissues were carefully removed from bone specimen before they were proceeded to experiments or archived in 70% EtOH.

For *μ*CT scan, long bone specimens were rehydrated with PBS and *μ*CT scan was performed. Long bone specimens were then retrieved from cylindrical vials, rehydrated with PBS and placed in decalcifying solution for 7 days before histological analysis. Histomorphometry was performed by TRAP staining on paraffin-embedded sections to visualize osteoclasts lining on the bone surface followed by counterstained with H&E.

### Assessment of cell-specific cytokine production

*In vitro* cell stimulation and intracellular flow analysis for MACS-purified lymphocytes: bone marrow- or spleen-derived CD4+ T-cells and CD8+ T-cells were purified by MACS according to the manufacturer's protocols. For stimulation of cytokine production, 500 000 purified cells were placed in modified complete RPMI media containing 5 *μ*g/ml brefeldin A, 50 ng/ml PMA, 0.5 *μ*g/ml ionomycin and cultured at 37 °C for 4 h. To assess cytokine production, stimulated cells were harvested into ice-old PBS, washed and fixed according to eBiosciences (San Diego, CA, USA) intracellular flow cytometry protocol before stained with anti-cytokine FACS antibodies and analyzed on flow cytometer.

### RNA isolation and qRT-PCR

Single mononucleated, red blood cell lysed cell suspension derived from bone marrow, spleen, thymus or lymph nodes or MACS-purified cells were lysed in Trizol reagent (Invitrogen, Carlsbad, CA, USA) for isolation of total RNA. cDNA synthesis was performed from 1 ug of total RNA with a mixture of random hexamers and poly-T oligos using Roche (Indianapolis, IN, USA) cDNA synthesis kit. To assess the abundance of mRNA transcripts for cytokine or transcription factors that mediate cytokine production, qPCR analysis was performed with SYBR Green master mix (Bio-Rad, Hercules, CA, USA) with primers corresponding to the gene of interest. GAPDH, tubulin or Cyclophilin A gene expression was monitored as endogenous control. All real-time PCR reactions were run on BioRad's CFX96 Real-Time System.

### Multiplex cytokine/chemokine profiling for serum

Mouse peripheral blood was collected in Microtainer serum separator tube (BD Bioscience, Franklin Lakes, NJ, USA) before centrifugation to obtain serum. All serum samples were archived in −80 °C freezer before being subjected to cytokine/chemokine measurement.

Multiplex cytokine/chemokine assay was performed according to the manufacturer's protocol (Millipore, St Charles, MO, USA). Briefly, 25 ul serum samples were loaded onto the 32-plex Mouse Cytokine/Chemokine Magnetic Bead Panel 96-well plate (MCYTOMAG-70 K-PX32). Following the incubation with 25 ul of magnetic beads at 4 °C overnight, biotin-conjugated antibodies and PE-conjugated streptavidin were subsequently added before the plate was processed with an automated washer and analyzed on a Bioplex system (Bio-Rad)

### *Ex vivo* osteoclast proliferation and differentiation assay

#### BrdU flow assay

After being cultured overnight on petridish with 1/50 diluted CMG, stromal cells were removed from WBM culture by re-plating suspension cells on tissue culture-treated plates to initiate macrophage culture. BrdU was added to macrophage culture with or without the presence of RANKL (50 ng/ml) for 4 days. For BrdU flow assay, cells were detached by Accutase and fixed with Foxp3 fix/perm buffer after washing with PBS. Fixed cells were permeabilized, digested with DNAase I, labeled with Alexa647-conjugated anti-BrdU Ab, washed and resuspended in the presence of 7-AAD before analyzing on a flow cytometer.

#### Assessment and quantification of osteoclast formation

After removal of adherent stromal cells, WBM cells were plated on 96-well microplates at a series of seeding densities from 200 000, 100 000, 50 000, 25 000, 12 500 and 6250 per well (or as otherwise mentioned). After stimulation with RANKL for 3–4 days, fixation and TRAP staining was performed to visualize multinucleated osteoclasts using the Leukocyte Acid Phosphatase kit from Sigma (St. Louis, MO, USA). Cultures without the presence of RANKL were served as the negative control.

### FACS-based profiling for hematopoietic progenitor cells, osteoclast progenitors and myeloid lineage cells

For assessment of hematopoietic progenitor cells in the bone marrow, WBMs were suspended in FACS buffer at the maximum concentration of 10 million cells per 100 ul and stained with biotin-conjugated lineage Ab cocktail (anti-B220, anti-CD3e, anti-Gr1, anti-Ter119 and anti-CD41) and HSC Ab cocktail (anti-Sca1 PerCP Cy5.5, anti-c-Kit APC eFluor 780, anti-CD34 FITC, anti-FLK2 APC, anti-CD48 PE Cy7, anti-CD150 PE and CD16/32 eFluor450) before red cell lysis. All FACS antibodies were purchase from either eBioscience, BioLegend (San Diego, CA, USA) or BD Bioscience (San Diego, CA, USA). Following incubation on ice for 45 min, Ab-labeled cells were washed twice with cold FACS buffer and either fixed with 2% PFA or immediately subjected to flow cytometric analysis.

### MACS and FACS sorting of bone marrow subpopulations for *ex vivo* osteoclastogenesis assay

To obtain lineage-negative CD11b^+^ and CD11b^-^ cells, WBM cells were red cell-lysed, first labeled with biotin-conjugated lineage antibodies and then incubated with anti-biotin magnetic microbeads to deplete lineage cells. Anti-CD11b microbeads was subsequently used to separate CD11b^+^ from CD11b^-^ cells. These MACS-purified cells were subjected to osteoclastogenesis assay as routinely done for WBMs or bone marrow macrophages, except they were seeded at a much lower series of density to obtain the optimal number of multinucleated osteoclast formation. MDSC isolation kit was used to obtain bone marrow cells that immuno-phenotypically resembled MDSCs.

For isolation of LSK HSCs, CMPs and GMPs, WBM cells were labeled with FACS antibodies as described above for progenitor analysis and sorted by either Sony Synergy (Sony Biotechnology, San Jose, CA, USA) or MoFlo sorter (Beckman Coulter, Indianapolis, IN, USA). These progenitor cells were sorted into ice-old culture alpha MEM supplemented with 20% FBS to maximize their survival rate. After sorting, cell counts were determined after resuspending cell pellets in a small volume of culture media before plating on 96-well microplates for osteoclastogenesis assay.

### Osteoclast founder cell assay

FACS-sorted HSCs, CMPs and GMPs were mixed with WBM cells at 1 : 9 ratio before subjecting to osteoclastogenesis assay. To test their sensitivity to M-CSF, recombinant M-CSF (Peprotech, Rocky Hill, NJ, USA) instead of CMG was used in the culture, and standard *ex vivo* osteoclastogenesis protocol was followed.

### Neutralizing Ab anti-M-CSF

Seven-day-old mice were i.p. injected with M-CSF neutralizing Ab (Bio X Cell, West Lebanon, NH, USA) twice a week for 3 weeks before killing for end-point analysis. Control mice were injected with equal volume of sterile PBS in parallel.

### Pathway inhibitors

Rapamycin working solution was prepared by diluting 10 mg/ml stock solution with 5% Tween80/5% PEG-400 by fivefold. Seven-day-old mice were then i.p. injected with 20 ug rapamycin through a 25 ul Hamilton syringe (Hamilton Co., Reno, NV, USA). Mice were re-administered five more times for the course of 3 weeks before end-point analysis. Control mice were injected with the vehicle solution.

### Western blot analysis and co-immunoprecipitation

For sample preparation, primary tissues (e.g., bone marrow, spleen, lymph nodes, thymus, peritoneal cavity) were processed into single mononuclear cell suspension after red blood cells were removed. To obtain protein lysates for western analysis, CLB buffer was used. For co-immunoprecipitation studies, RIPA buffer was used instead. Lamieli sample buffer (5 × ) was added to each protein lysate with *β*-mercaptoethanol before boiling for 5 min for protein denaturation and reduction. Samples were then resolved on 8% SDS-PAGE gels, blotted on PVDF membranes using Bio-Rad's Trans-Blot Turbo system before probes with antibodies.

### Luciferase NF-*κ*B reporter assay

293T cells were co-transfected with Luciferase reporter and Renillia luciferase plasmids with pcDNA plasmids carrying NEMO and Foxp3 cDNA. Renilla luciferase activity was used as internal loading control. Luficerase activity was assessed by Promega's Stop-and-Go system. For each sample, Luciferase activity was normalized by Renilla luciferase activity.

## Abbreviations

Sf: scurfy
WT: wild type
Op: osteopetrotic
WBM: whole bone marrow
RANKL: RANK ligand
LSK: lineage negative Sca-1 positive c-Kit positive
MPs: myeloid precursors
LT-HSCs: long-term hematopoietic stem cells
ST-HSCs: short-term hematopoietic stem cells
CMPs: common myeloid progenitors
GMPs: granulocyte/macrophage progenitors
