# Supplementary material for: Myeloid lineage skewing due to exacerbated NF-κB signaling facilitates osteopenia in Scurfy mice
Source: Cell Death Dis. 2015 Apr 16;6(4):e1723–. doi: 10.1038/cddis.2015.87 (PMC4650554; doi:10.1038/cddis.2015.87)
Supplement: Supplementary Information [file cddis201587x1.doc]

**Supplementary Material:**

**SUPPLEMENTARY FIGURES:**

**Figure S1. Expression of osteoclast markers in scurfy WBM cultures and expression of NF-kB genes in scurfy T helper cells**. (A) Total RNA was isolated after 4 days of RANKL stimulation, cDNA was synthesized and followed by qPCR analysis. Expressions of osteoclastogenesis markers were normalized by GAPDH in delta/delta CT format. (B) Densitometric analysis of RelA and NEMO protein expression in MACS purified CD4^+^ T_H_ cells shown in **Fig 2A**. (C) qRT-PCR analysis of RelA and NEMO mRNA expression in CD4^+^ T_H_ cells. Statistics for bar graphs in A: n= 6 per group; *p<0.005 vs. WT-RL and **p<0.002 vs. WT+RL. In B: n=4 per group; *p<0.05. In C: n=3 per group; *p<0.02 by Student T-test

**Figure S2. Increased NEMO-expressing cell population in scurfy bone marrow.** Flow cytometric analysis with (A) myeloid marker CD11b and progenitor marker c-kit to identify NEMO-expressing cells, and with (B) myeloid marker CD11b and Gr-1 on NEMO positive cells.

**Figure S3. Dysregulation of NEMO expression in scurfy mice** (A) NF-κB activation attenuated by forced expression. 293T cells were co-transfected with luciferase reporter and plasmid together with *Foxp3* plasmid. After 48 hrs, transfected cells were harvested, lysed, and subjected to luciferase activity measurement by luminometer with luciferase substrate added. Cells without transfected with the *Foxp3* plasmid was served as control. (A) Increased NEMO protein expression in scurfy lymph nodes (B) Protein lysates of mononuclear cells derived from 4 scurfy mice with 2 WT littermates were analyzed by Western blot probed with anti-NEMO antibody. (C) Lymph node derived mononuclear cells were also analyzed for NEMO expression by intracellular flow cytometry. MFI of FITC was depicted by histogram similarly to Figure 2B and bar graphed. (D) Increased NEMO protein expression in the thymus of scurfy. (Intracellular flow analysis was performed on thymus-derived cells. (E) Increased NEMO protein expression in scurfy bone marrow CD11b^-^ MACS fraction. MACS was performed on WBMs derived from 2 scurfy and 2 WT controls using CD11b microbeads to separate CD11b^+^ an CD11b^-^ cells. After MACS, cells fractions were lysed to obtain protein for Western analysis. (F) No change in NEMO protein expression in the peritoneal cavity of scurfy mice. Western analysis for NEMO protein expression was also performed on mononuclear cells harvested by peritoneal cavity perfusion. Statistics for A: n= 3 per group; *p<0.05 vs. +TNFα with 0 μg of pcDNA-mFoxp3 and **p<0.0005 vs. +RANKL/LPS with 0 μg of pcDNA-mFoxp3 by Student T-test. For all other bar graphs: n= 6 for WT and Sf *p<0.05 vs. WT by Student T-test.

**Figure S4. Hypercytokinemia** **in scurfy mice.** Multiplex analysis was performed using the Milliplex cytokine/chemokine panel on serum samples derived from 4 scurfy mice together with 2 WT littermate controls. 25 μl of each serum sample was used in the assay. Statistics for all scatter plots: n= 2 for WT and 3 for Sf; *p<0.05 and **p<0.005 by Student T-test.

**Figure S5. Hyperproliferation of scurfy bone marrow cells in response to M-CSF.** Alamar Blue cell proliferation assays were performed on WBM cultures without (A) or with (B) RANKL in a time course manner. n= 4 per group; *p<0.05 by Student T-test.

**Figure S6.** **Myeloid cells exhibiting MDSC immunotypes in scurfy BM were not potent osteoclast precursors.** (A) Yield of MACS isolation of Gr-1^high^Ly6G^+^ and Gr-1^dim^Ly6G^-^ MDSCs. Two MSDC subsets were purified from WT and scurfy BM using Miltenyi’s MDSC isolation kit (B) (B-E) None of the MACS purified MDSC subsets were oscteoclastogenic. Ex vivo oscteoclastogenesis assay performed with Gr-1^high^Ly6G^+^ and Gr-1^dim^Ly6G^-^ MDSCs (B and C). Whole bone marrow (D) and cells devoid of the two MDSC populations (E) were used as control. Statistics for A: n= 3 for each group; *p<0.007 and **p<0.0001 by Student T-test. Scale bars: 200 μm in B-E.

Figure S7. **Cell sorting scheme for the isolation of progenitor cells LSKs and MPs for ostoclastogenic founder cell assay.** FACS antibody-stained, red cell-lysed whole bone marrow samples were first gated for live cells by FSC/SSC, followed by FSC-A/FSC-W gate for doublet discrimination. After eliminating lineage positive cells, lineage negative cells were further gated by Sca-1/c-kit to obtain Sca-1^+^c-kit^+^cells (LSKs) and by CD11b/c-kit to obtain CD11b^lo^c-kit^+^ cells (MPs). Further examination of MP progenitor cells also showed their low Gr-1 expression (data not shown).

**Figure S8: scurfy derived LSK HSCs are hypersensitive to M-CSF**. (A) 100,000 WBMs (controls) and 5,000 FACS isolated LSK HSCs were subjected to ex vivo osteoclastogenesis assay under a serial dosage of M-CSF. (B) PU.1 intracellular flow analysis was performed on WT and Sf derived bone marrow cells with anit-PU.1 and anti-NEMO antibodies in addition to antibodies required for the identification of LSK progenitor cells. Scale bars: 200 μm in A.

**Figure S9. Increased frequency of CD150-CD48+ LSKs (MPPs) in scurfy BM was corrected by *in vivo* administration of M-CSF neutralizing antibody.** WBMs were harvested from moribund M-CSF neutralizing antibody and vehicle treated mice, red cell lysed and stained with FACS antibodies for progenitor analysis. Based on CD150 and CD48 surface marker expression LSK cells were further sub-gated into CD115+CD48- (LT-HSCs), CD140^+^CD48^+^ (ST-HSCs), and CD150^-^CD48^+^(MPPs) primitive hematopoietic progenitor cell populations. Statistics for bar graph: n= 3 for WT group and n=4 for Sf group; *p<0.05 by Student T-test.
