# Supplementary figures and images for: Myeloid lineage skewing due to exacerbated NF-κB signaling facilitates osteopenia in Scurfy mice
Source: Cell Death Dis. 2015 Apr 16;6(4):e1723–. doi: 10.1038/cddis.2015.87 (PMC4650554; doi:10.1038/cddis.2015.87)

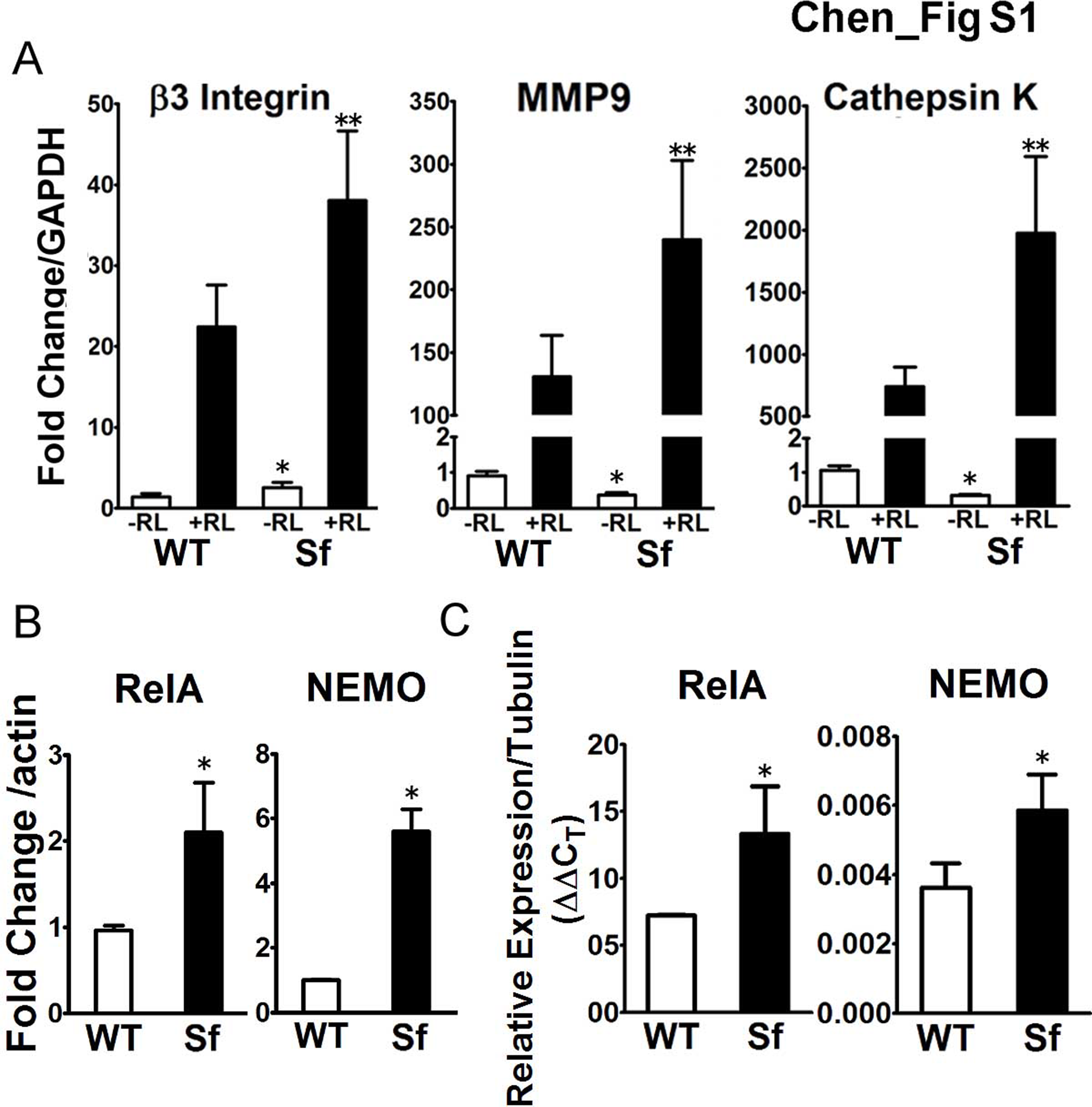

Supplement: Supplementary Figure 1 [file cddis201587x2.tif]

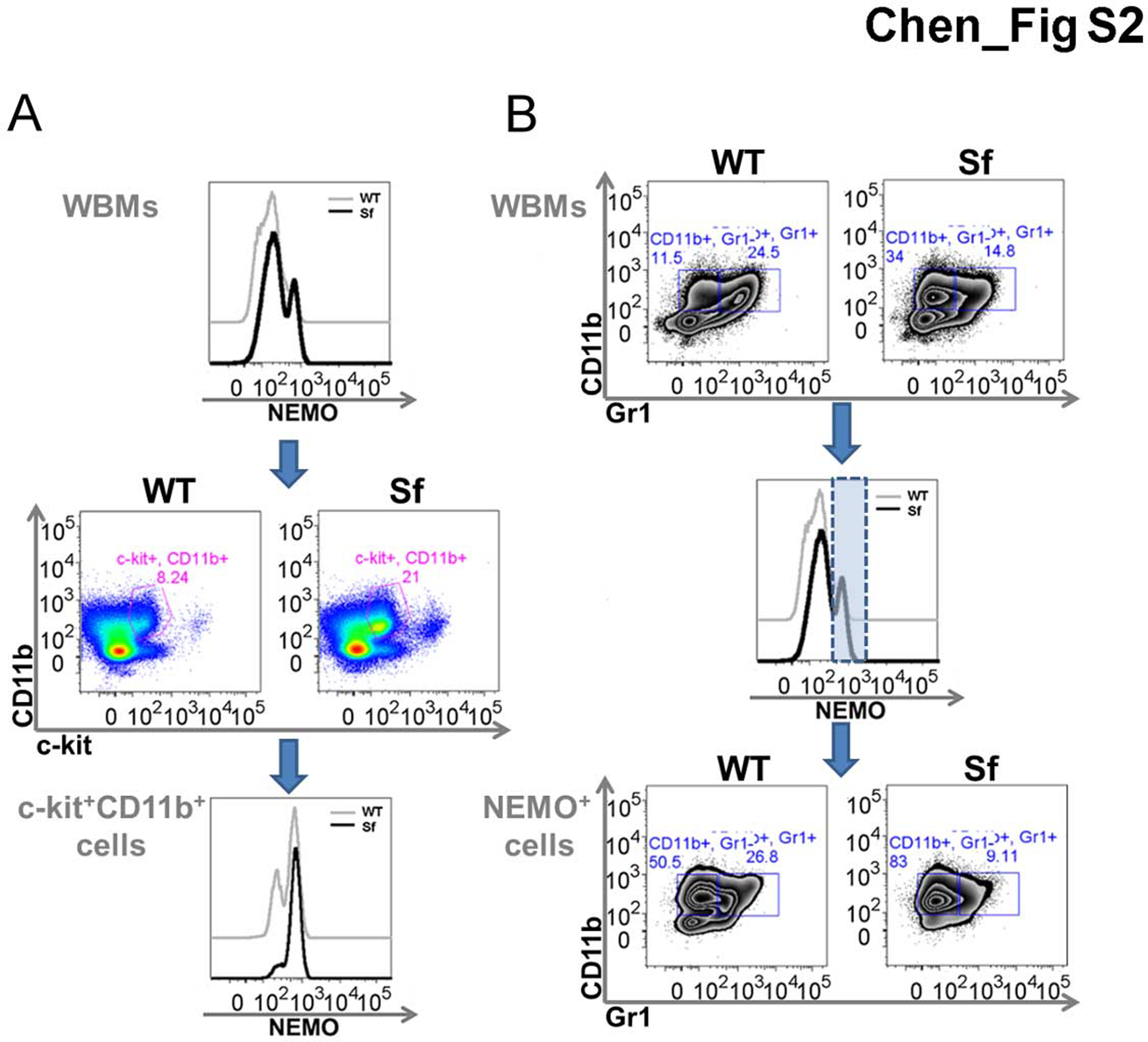

Supplement: Supplementary Figure 2 [file cddis201587x3.tif]

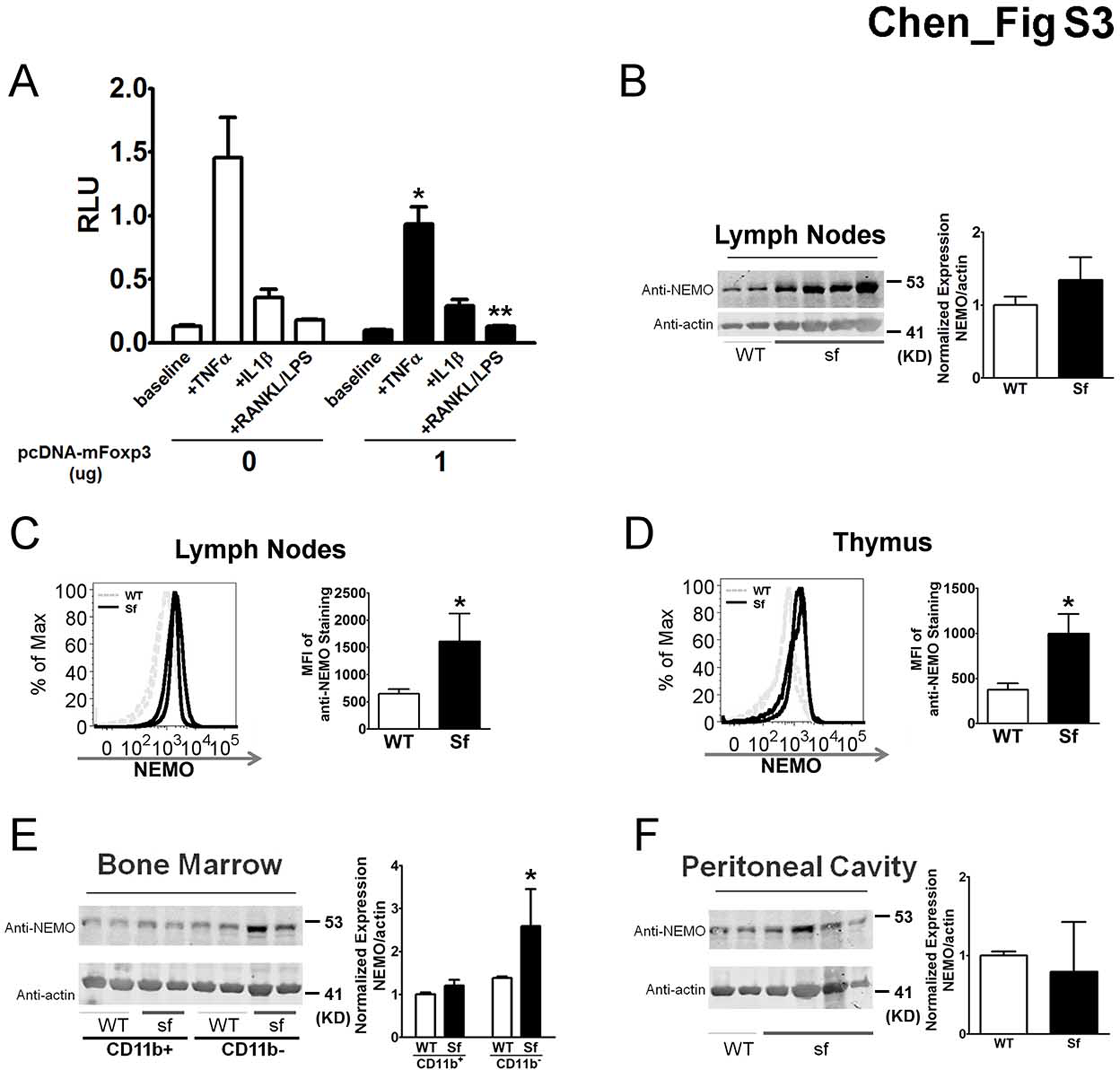

Supplement: Supplementary Figure 3 [file cddis201587x4.tif]

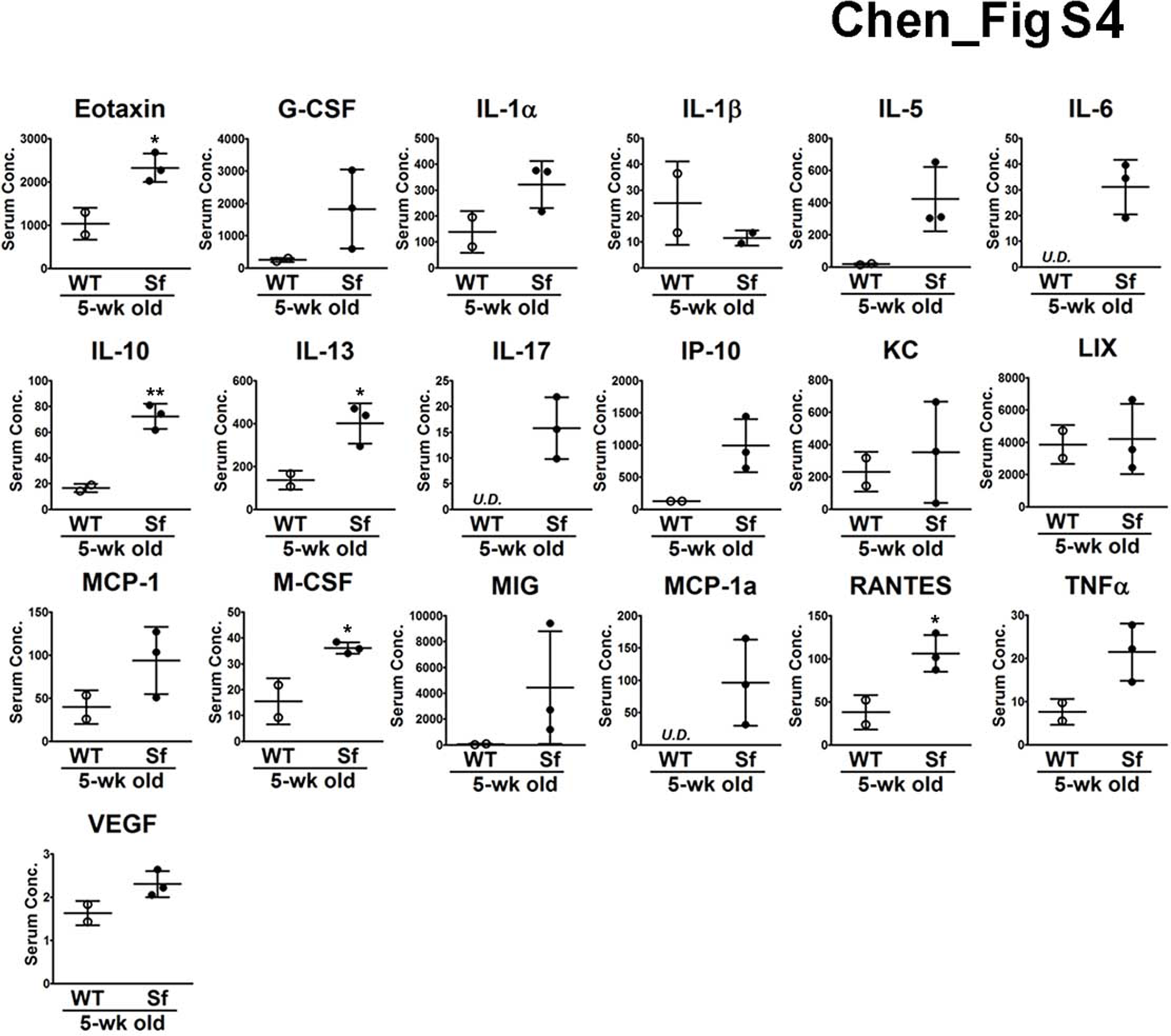

Supplement: Supplementary Figure 4 [file cddis201587x5.tif]

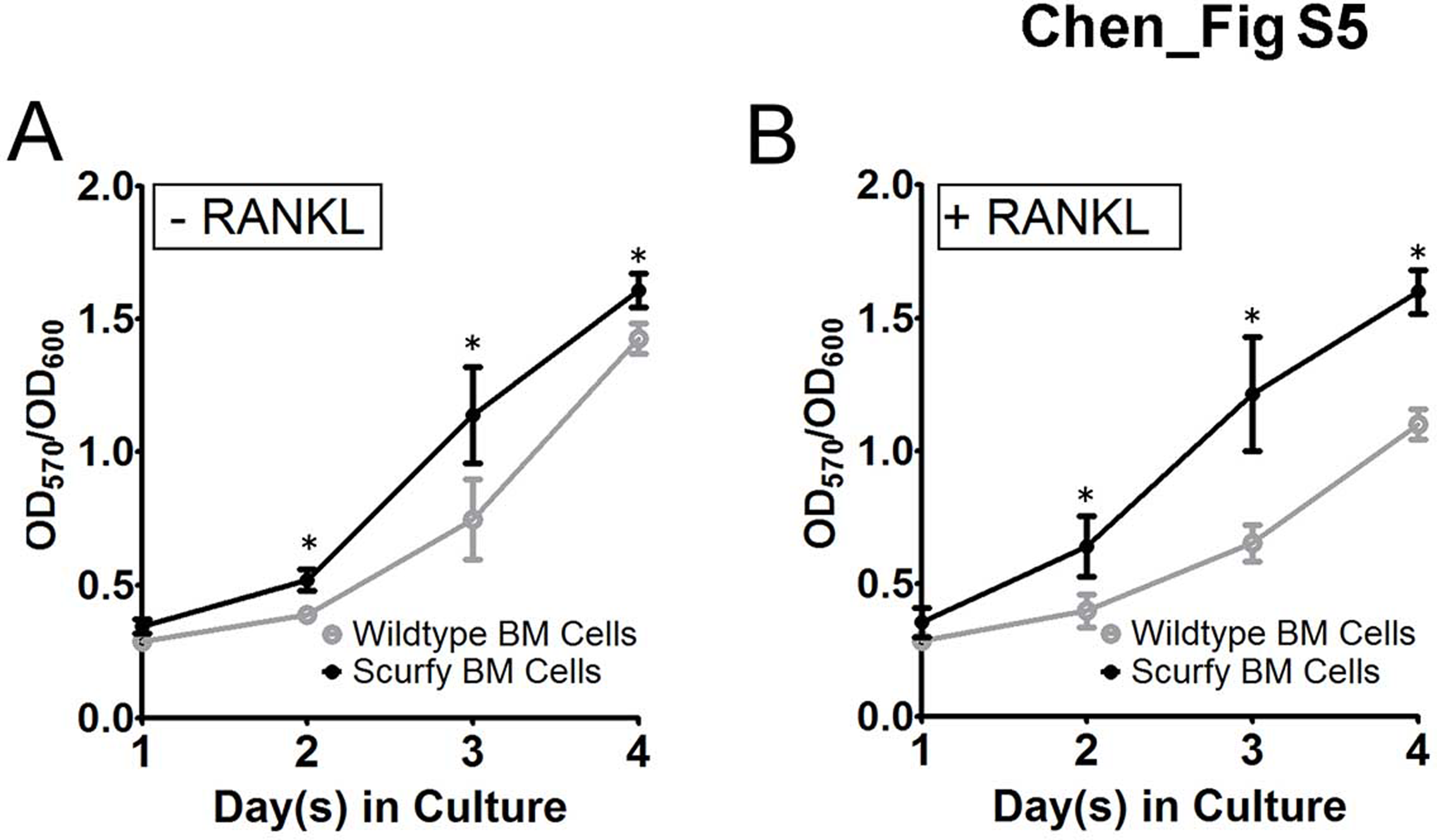

Supplement: Supplementary Figure 5 [file cddis201587x6.tif]

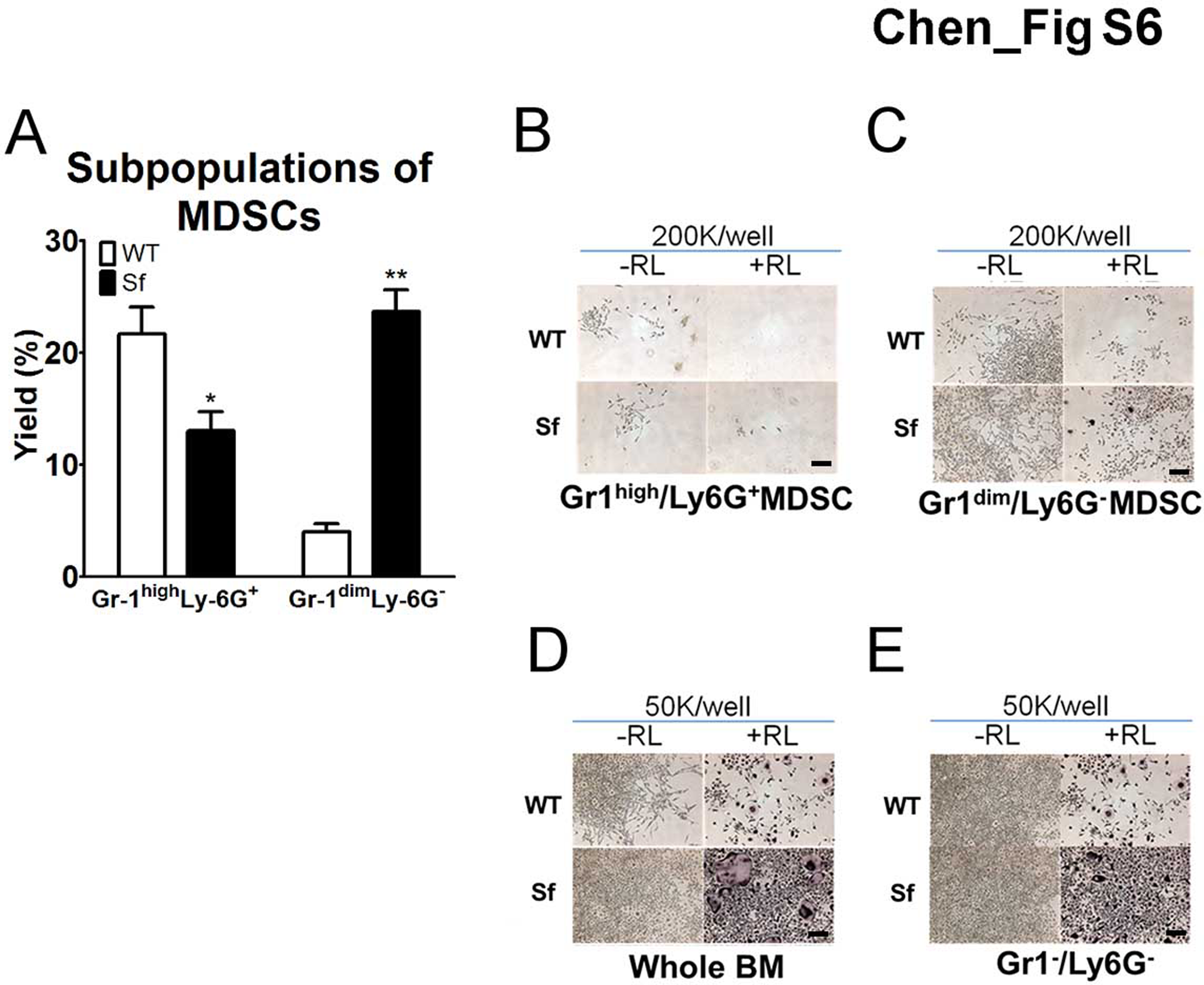

Supplement: Supplementary Figure 6 [file cddis201587x7.tif]

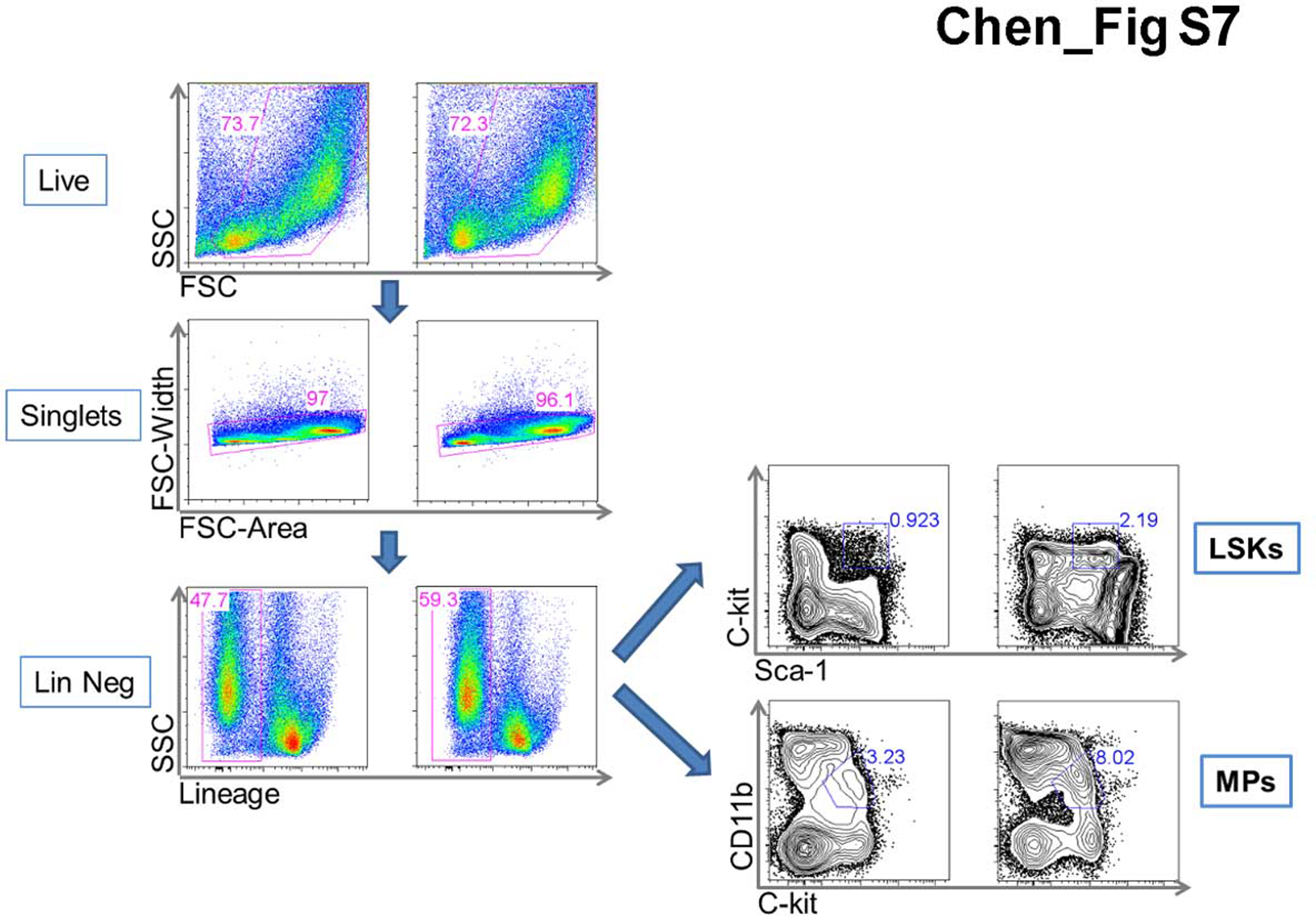

Supplement: Supplementary Figure 7 [file cddis201587x8.tif]

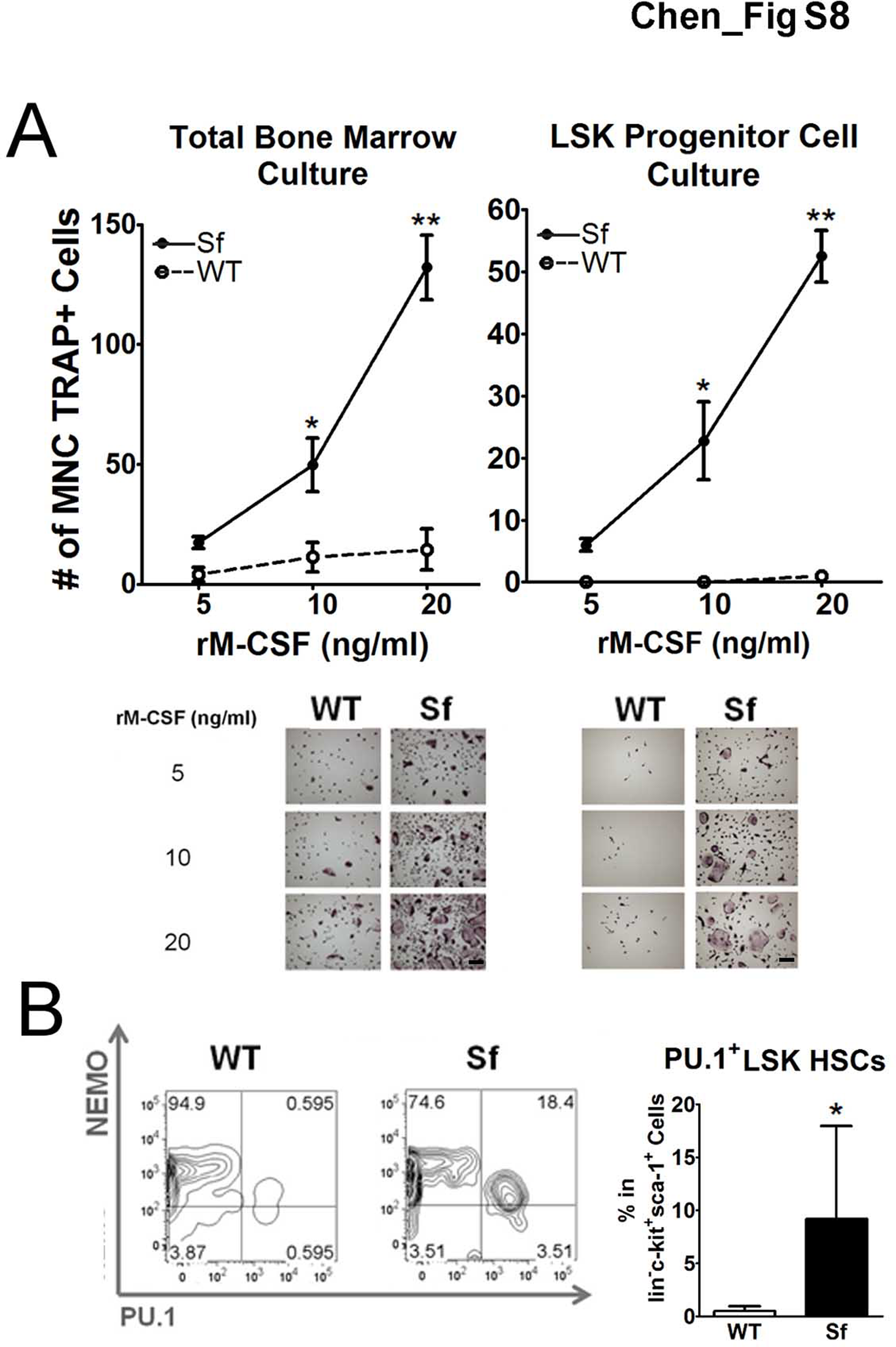

Supplement: Supplementary Figure 8 [file cddis201587x9.tif]

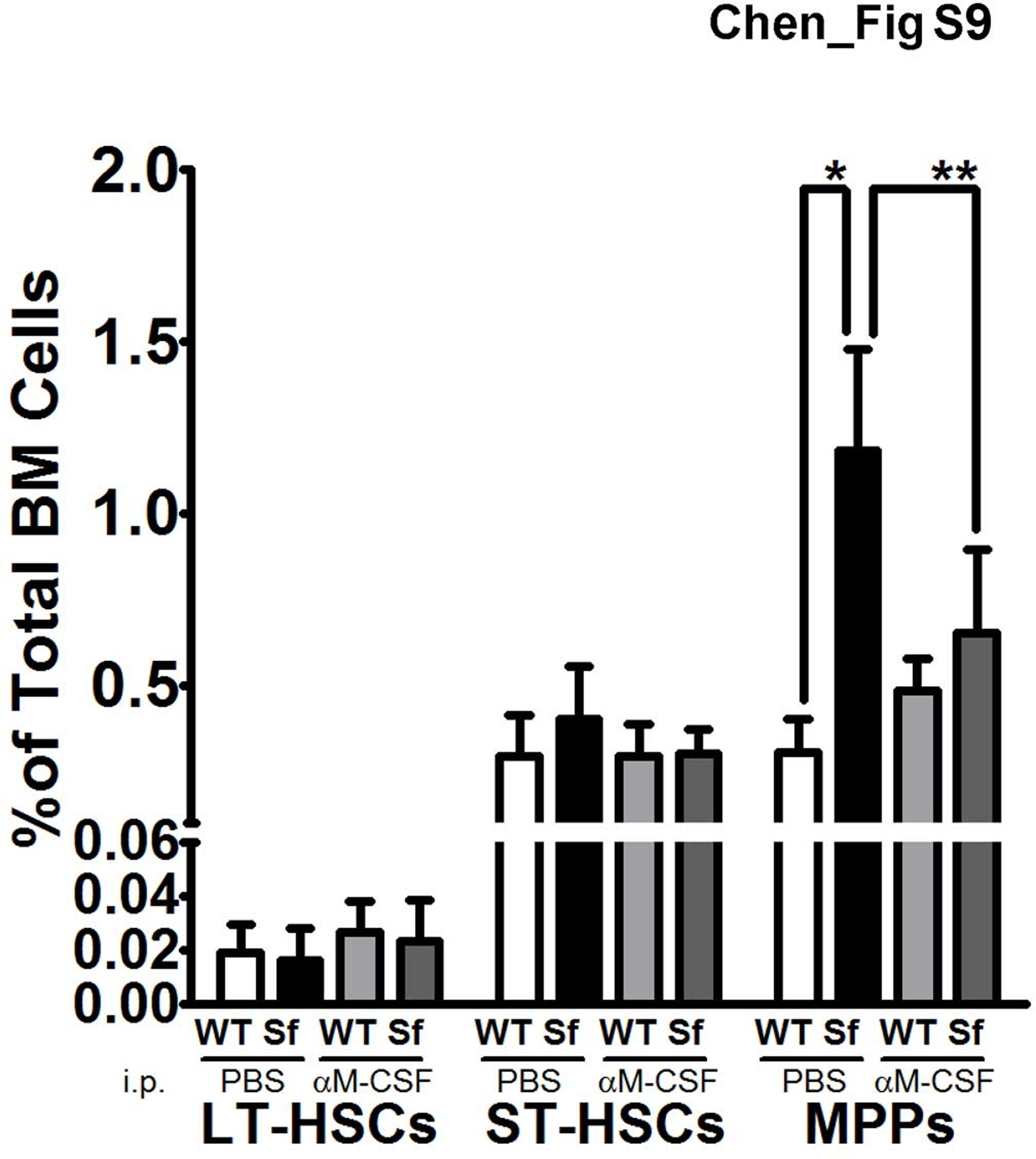

Supplement: Supplementary Figure 9 [file cddis201587x10.tif]
